# Necroptosis Induced by Delta-Tocotrienol Overcomes Docetaxel Chemoresistance in Prostate Cancer Cells

**DOI:** 10.3390/ijms24054923

**Published:** 2023-03-03

**Authors:** Marina Montagnani Marelli, Giangiacomo Beretta, Roberta Manuela Moretti

**Affiliations:** 1 Department of Pharmacological and Biomolecular Sciences, Università degli Studi di Milano, 20133 Milan, Italy; 2 Department of Environmental Science and Policy, Università degli Studi di Milano, 20133 Milan, Italy

**Keywords:** necroptosis, delta-tocotrienol, prostate cancer, docetaxel, chemoresistance

## Abstract

Prostate cancer (PCa) represents the fifth cause of cancer death in men. Currently, chemotherapeutic agents for the treatment of cancers, including PCa, mainly inhibit tumor growth by apoptosis induction. However, defects in apoptotic cellular responses frequently lead to drug resistance, which is the main cause of chemotherapy failure. For this reason, trigger non-apoptotic cell death might represent an alternative approach to prevent drug resistance in cancer. Several agents, including natural compounds, have been shown to induce necroptosis in human cancer cells. In this study we evaluated the involvement of necroptosis in anticancer activity of delta-tocotrienol (δ-TT) in PCa cells (DU145 and PC3). Combination therapy is one tool used to overcome therapeutic resistance and drug toxicity. Evaluating the combined effect of δ-TT and docetaxel (DTX), we found that δ-TT potentiates DTX cytotoxicity in DU145 cells. Moreover, δ-TT induces cell death in DU145 cells that have developed DTX resistance (DU-DXR) activating necroptosis. Taken together, obtained data indicate the ability of δ-TT to induce necroptosis in both DU145, PC3 and DU-DXR cell lines. Furthermore, the ability of δ-TT to induce necroptotic cell death may represent a promising therapeutical approach to overcome DTX chemoresistance in PCa.

## 1. Introduction

Prostate cancer (PCa) is the second most common solid tumor in men and the fifth cause of cancer mortality [1,2]. Patients with early-stage PCa are usually managed with active surveillance, radiation or surgery. Most patients are first treated through androgen deprivation therapy (ADT), but almost all of them develop resistance to this therapeutic approach, progressing to castration-resistant prostate cancer (CRPC) for which new effective therapeutic approaches are required [3,4]. Currently, different approved systemic therapies can prolong the survival of patients with CPRC. Among these therapies, docetaxel (DTX) is the approved choice for prostate cancer treatment in combination with prednisone [5,6,7,8]. However, despite the efficacy benefits achieved with DTX-based treatment, approximately half of all patients do not respond, while some others eventually develop DTX-resistance [9].

Multiple factors contribute to DTX-resistance, such as mutations or overexpression in the tubulin gene [10], androgen receptor activation [11], mutations or overexpression of ATP-binding cassette (ABC) or multidrug resistance (MDR) transporters [12,13], autophagy activation [14,15], resistance to apoptosis [16], acquisition of epithelial to mesenchymal transition (EMT) [17,18,19] and expansion of cancers stem cells (CSC) [20,21].

For these reasons, it is extremely important to explore new potential therapeutic strategies to overcome chemotherapy resistance in prostate cancer. Recent studies exploring the antitumor effects of natural compounds and their extracts have demonstrated the potential efficacy in governing drug resistance in cancer cells activating alternative regulated cell death (RCD) [22,23]. Although the most studied RCD is apoptosis, many other processes, such as autophagy, pyroptosis, ferroptosis and necroptosis, represent different forms of cell death that could be triggered by alternative therapies when cancer cells develop resistance to apoptosis. Tocotrienols (TTs), belonging to the vitamin E family together with tocopherols, present four isoforms (α-, β-, γ- and δ-) characterized by a chromanol ring linked to an isoprenoid side chain at the C2 position; this chain is saturated in tocopherols and unsaturated in TTs. Several studies have pointed out that γ- and δ-TT specifically exert an antitumoral activity affecting proliferation, metastasis and angiogenesis in different types of tumors [24,25,26,27,28].

In PCa TTs reduce proliferation [29] and activate apoptosis [30,31] modulating the expression of different targets, such as nuclear factor-kB (NF-kB) [32] and multiple pathways [31,33,34]. In CRPC, γ-TT is also able to downregulate the expression of the cancer stem cell (CSC) markers CD133/CD44 interfering with the spheroid formation [35].

Recently, we have reported that δ-TT exhibits antitumor activity by triggering apoptosis involving ER stress, autophagy and paraptosis in CRPC cell lines [34].

Necroptosis is a type of caspase-independent programmed cell death that shows morphological features of necrosis, and it is characterized by increased cellular volume, organelle shrinkage and membrane permeability [36]. The triggering of the necroptotic process depends on the activation of receptor-interacting serine/threonine protein kinase (RIP)1/3 and mixed lineage kinase domain-like (MLKL). The canonical necroptosis pathway is initiated by the kinase activity of RIP1 followed by recruitment and activation of RIP3 (necrosome complex), which in turn activates MLKL through serin phosphorylation. The activation of MLKL, executioner of necroptosis, promotes its oligomerization and translocation from the cytosol on cellular membranes, including the plasma membrane, where it induces loss of membrane integrity, which results in the influx of sodium and calcium ions, which determine necroptosis execution [37]. Recent studies reveal that necroptosis could represent an alternative strategy for killing cancer cells when they have established resistance to apoptotic cell death [36,38].

The current study aims to explore the involvement of necroptosis in the anticancer activity of δ-TT in two CRPC cell lines (DU145 and PC3). The results in this manuscript indicate that δ-TT induces necroptosis through RIP1 and MLKL involvement causing necroptotic cell death in CRPC cells.

On the other hand, δ-TT treatment sensitizes DU145 cells to DTX and induces cell death in DU145-derived DTX-resistant (DU-DXR) cells generated in our laboratory through the involvement of a necroptotic process.

Taken together, the results of this study suggest that necroptosis-based cancer therapy using δ-TT may be an alternative way of tackling cancer cells that acquired chemotherapy resistance.

## 2. Results

### 2.1. δ-TT Induces Prostate Cancer Cell Death

**Figure 1:**
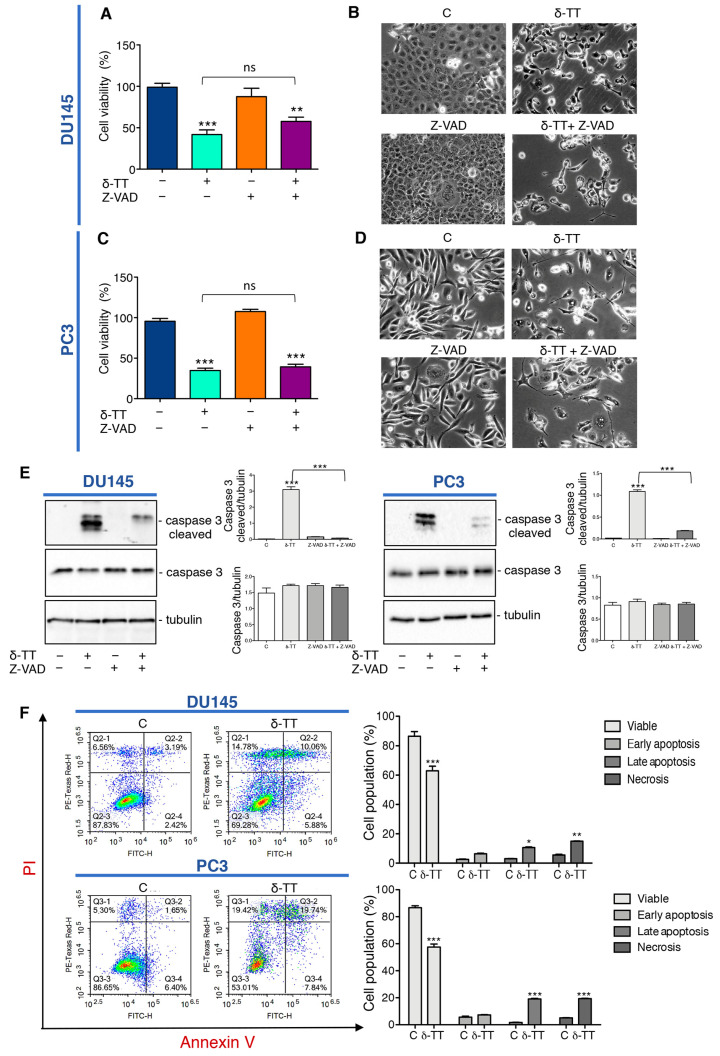
δ-Tocotrienol (δ-TT) induces cell death in prostate cancer cells. (**A**) Cell viability analysis after δ-TT treatment (20 μg/mL, 24 h) in combination with or without pan-caspase inhibitor carbobenzoxy-valyl-alanyl-aspartyl-[O-methyl]- fluoromethylketone (Z-VAD) pretreatment (50 μM, 4 h) was determined by MTT (3-(4,5)-dimethylthiazol-2-yl-2,5-diphenyltetrazolium bromide) assay in DU145 cells. Six independent biological samples for each condition were analyzed (*n* = 6); bar graph represents the mean relative cell viability ± SD. Statistical analysis was performed using one-way ANOVA followed by Bonferroni post-hoc test (** = *p* < 0.01 vs. C; *** *p* < 0.001 vs. C). (**B**) Morphological analysis of DU145 cells after δ-TT treatment (20 μg/mL, 24 h) in combination with or without Z-VAD pretreatment (50 μM, 4 h) was analyzed by phase-contrast microscopy. (**C**) Cell viability analysis after δ-TT treatment (20 μg/mL, 24 h) in combination with or without pan-caspase inhibitor Z-VAD pretreatment (50 μM, 4 h) was determined by MTT assay in PC3 cells. Six independent biological samples for each condition were analyzed (*n* = 6); bar graph represents the mean relative cell viability ± SD. Statistical analysis was performed using one-way ANOVA followed by Bonferroni post-hoc test (*** = *p* < 0.01 vs. C). (**D**) Morphological analysis of PC3 cells after δ-TT treatment (20 μg/mL, 24 h) in combination with or without Z-VAD pretreatment (50 μM, 4 h) performed by phase-contrast microscopy. (**E**) The expression of caspase 3 and caspase 3 cleaved were analyzed by Western blot after treatment with δ-TT (20 μg/mL, 24 h) in combination with or without Z-VAD (50 μM) in DU145 and PC3 cells. Tubulin was used as loading control. Three independent experiments for each condition were analyzed; bar graph represents the mean optical density ± SD. Statistical analysis was performed using one-way ANOVA followed by Bonferroni post-hoc test (*** *p* < 0.001). (**F**) Flow cytometric analysis by Annexin-FITC/PI double staining was performed after δ-TT treatment (20 μg/mL, 48 h) in both DU145 and PC3 cells. Three independent biological samples for each condition were analyzed (*n* = 3); bar graph represents the mean ± SD. Statistical analysis was performed using Student’s test (* *p* < 0.05 vs. C; ** *p* < 0.01 vs. C; *** *p* < 0.001 vs. C).

We previously demonstrated that δ-TT induces cytotoxicity in CRPC cell lines triggering apoptosis through activation of ER stress and autophagy, and the abrogation of apoptosis by the pan-caspase inhibitor Z-VAD-FMK (Z-VAD) significantly, but not completely, reverted the cytotoxic effect of δ-TT (15 μg/mL) [34]. Here we analyzed the effect of a higher dose of δ-TT (20 μg/mL) on cellular viability of DU145 and PC3 cell lines in the presence of Z-VAD. The results summarized in Figure 1A,C show that this dose of δ-TT significantly induces a cytotoxic effect in both cell lines, but surprisingly the treatment with Z-VAD does not significantly reverse the δ-TT cytotoxicity. In addition, as shown in the Figure 1B,D, DU145 and PC3 cells treated with δ-TT developed morphological changes (small vacuoles and rounded shape) compared with the untreated cells and Z-VAD in combination with δ-TT, which did not significantly counteract the phenotype δ-TT induced. However, as shown Figure 1E, the increase of caspase 3 cleaved induced by δ-TT, is significantly reversed by Z-VAD, revealing that apoptosis is involved in δ-TT cytotoxicity. Globally these results could suggest that, when apoptosis was chemically inhibited, this dose of δ-TT exerted cytotoxicity triggering another type of cell death. Concurrently, FACS analysis by Annexin-FITC/Propidium Iodide (PI) double staining showed that the treatment with δ-TT (20 μg/mL) for 48 h induced an accumulation of cells not only in late apoptosis, but also in necrosis (14.78% in DU145 and 19.42% in PC3 cells) in respect to control cells (6.56% in DU145 and 5.30% in PC3 cells) indicating that another type of cell death other than apoptosis occurred (Figure 1F). These results demonstrate that the cytotoxic action of δ-TT is not only attributable to apoptosis activation, but a necrotic phenomenon could also be involved.

### 2.2. δ-TT Induces Necroptosis Machinery in Prostate Cancer Cells

**Figure 2:**
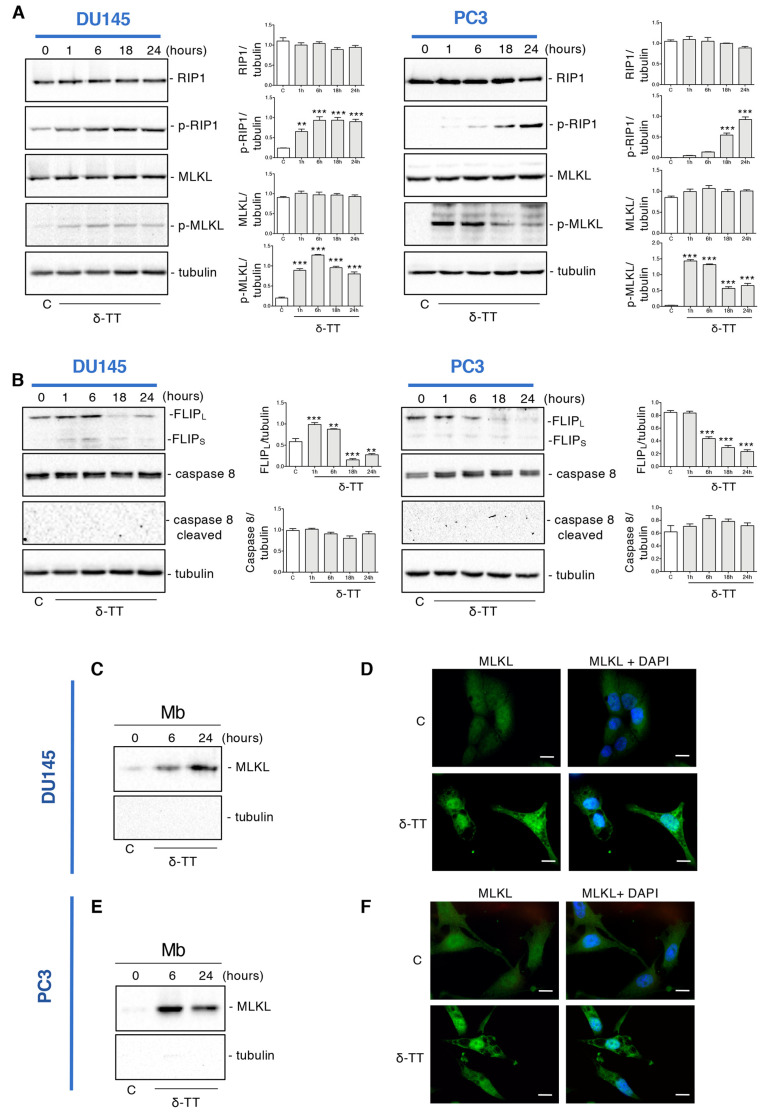
δ-TT induces necroptosis machinery in prostate cancer cells. (**A**) The expression of proteins involved in necroptosis activation (RIP1, p-RIP1, MLKL, p-MLKL) was analyzed by Western blot analysis after treatment with δ-TT (20 μg/mL) from 1 h to 24 h in both DU145 and PC3 cells. Tubulin was used as loading control. Three independent experiment for each condition were analyzed; bar graph represents the mean optical density± SD. Statistical analysis was performed using one-way ANOVA followed by Dunnett’s post-hoc test (** *p* < 0.01 vs. C; *** *p* < 0.001 vs. C). (**B**) The expression of FLIP_L_, FLIP_S_, caspase-8 and caspase-8 cleaved were analyzed by Western blot after treatment with δ-TT (20 μg/mL) from 1 h to 24 h in both DU145 and PC3 cells. Tubulin was used as loading control. Three independent experiments for each condition were analyzed; bar graph represents the mean optical density± SD. Statistical analysis was performed using one-way ANOVA followed by Dunnett’s post-hoc test (** *p* < 0.01 vs. C; *** *p* < 0.001 vs. C). (**C**) The expression of MLKL was evaluated in membrane preparations (Mb) after treatment with δ-TT (20 μg/mL) for 6 h and 24 h in DU145 cells. Analysis of tubulin expression demonstrate the lack of cytosolic contamination in membrane preparations. Three independent experiments for each condition were analyzed. (**D**) Analysis of MLKL localization was carried out by immunofluorescence after treatment with δ-TT (20 μg/mL, 24 h) in DU145 cells. Nuclei were stained by DAPI. Scale bar, 20 μm. (**E**) The expression of MLKL was evaluated in membrane preparations (Mb) after treatment with δ-TT (20 μg/mL) for 6 h and 24 h in PC3 cells. Analysis of tubulin expression demonstrate the lack of cytosolic contamination in membrane preparations. Three independent experiments for each condition were analyzed. (**F**) Analysis of MLKL localization was carried out by immunofluorescence after treatment with δ-TT (20 μg/mL, 24 h) in PC3 cells. Nuclei were stained by DAPI. Scale bar, 20 μm.

To clarify if the δ-TT-induced necrosis could be a regulated phenomenon, such as necroptosis, the expression of necroptosis biomarkers was examined by Western blotting (WB). The necroptosis cascade activation is mediated by the interaction of RIP1, RIP3 and MLKL proteins in their phosphorylated form [37]. Figure 2A shows that in both cell lines δ-TT (20 μg/mL) led to an increase of p-RIP1 and the downstream target p-MLKL in a time-dependent manner. Necroptosis induction also involves other molecules that could complex with RIP1, such as caspase 8 and FLICE-like inhibitory protein (FLIP). FLIP has mainly two isoforms: long (FLIP_L_) and short (FLIP_S_) [39]. FLIP_S_ promotes necroptosis, while FLIP_L_ can act as an anti-necroptotic molecule [40]. Indeed, when active caspase 8 heterodimerizes with FLIP_L_, it can cleave RIP1 and RIP3 leading to necrosome disassembling and triggering apoptosis. On the other hand, when caspase 8 is inactive, RIP1 interacts with RIP3 and triggers necroptosis [41]. Figure 2B shows that δ-TT decreased FLIP_L_ expression at 18 h and 24 h in DU145 cells and from 6 h to 24 h in PC3 cells. Instead, the FLIP_S_ isoform appeared only slightly increased after δ-TT treatment in DU145 cells, while it did not change in PC3 cells. Furthermore, consistent with the activation of necroptosis, δ-TT treatment did not cleave caspase 8 (Figure 2B).

Since the translocation of MLKL from cytosol to membranes is a determining event for necroptotic activation, we analyzed the expression of this protein in membrane preparations and its cellular localization by immunofluorescence.

Figure 2C shows that following δ-TT treatment (20 μg/mL), the quantity of MLKL in the plasma and internal membranes increased both at 6 h and 24 h in DU145 cells, compatibly with its translocation from the cytosol to the membranes. Likewise, immunofluorescence assays showed a diffused cytoplasmatic MLKL fluorescence in control cells, whereas in δ-TT-treated cells (20 μg/mL, 24 h), MLKL was localized preferentially at the membrane level (Figure 2D). The same observations were obtained in PC3 cells (Figure 2E,F).

Globally, these results indicate that δ-TT induces the activation of necroptosis in CRPC cells involving RIP1 and MLKL.

### 2.3. Inhibition of Necroptosis Prevents δ-TT Prostate Cancer Cell Death

**Figure 3:**
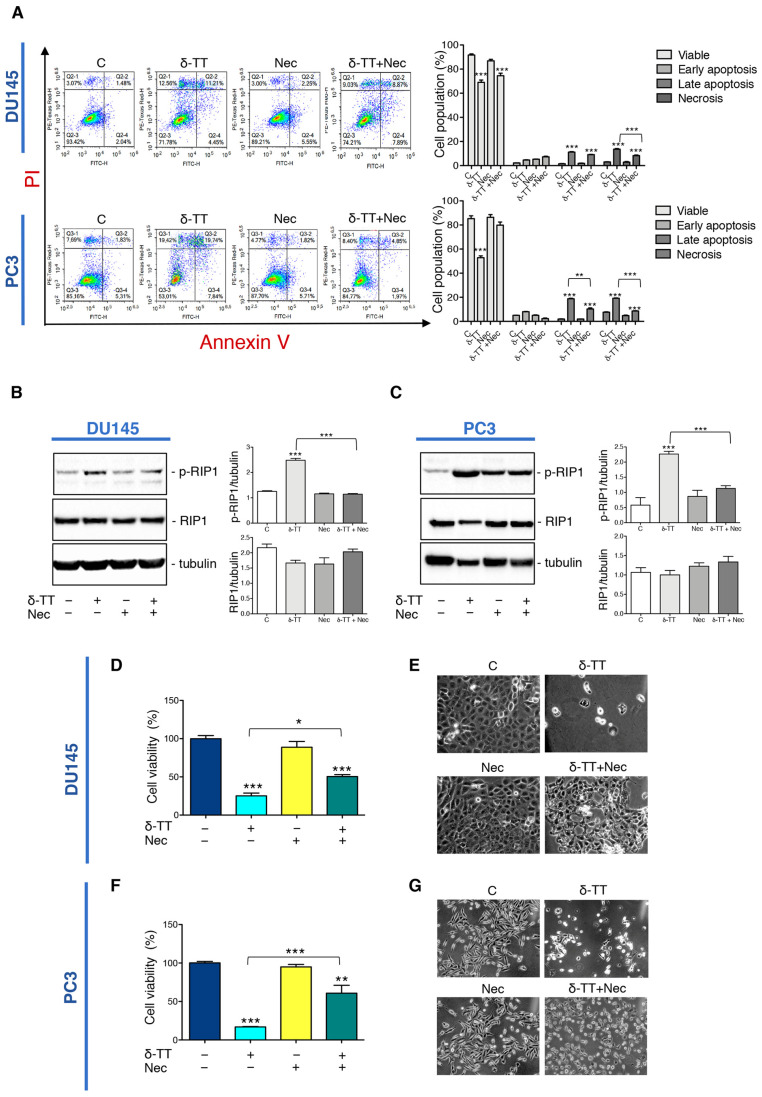
Inhibition of necroptosis prevents δ-TT prostate cancer cell death. (**A**) Flow cytometric analysis by Annexin-FITC/PI double staining was performed after δ-TT treatment (20 μg/mL, 48 h) in combination with necrostatin-1 (Nec) (50 μM) in both DU145 and PC3 cells. Three independent biological samples for each condition were analyzed (*n* = 3); bar graph represents the mean ± SD. Statistical analysis was performed using one-way ANOVA followed by Bonferroni post-hoc test (** *p* < 0.01; *** *p* < 0.001). (**B**) The effect of δ-TT (20 μg/mL, 24 h) in the presence of Nec (50 μM) was evaluated on p-RIP1 and RIP1 expression in DU145 cells. Tubulin was used as loading control. Three independent experiments for each condition were analyzed; bar graph represents the mean optical density± SD. Statistical analysis was performed using one-way ANOVA followed by Bonferroni post-hoc test (*** *p* < 0.001). (**C**) Effect of treatment with δ-TT (20 μg/mL, 24 h) in presence of Nec (50 μM) on RIP1 and p-RIP1 expression in PC3 cells. Tubulin was used as loading control. Three independent experiments for each condition were analyzed; bar graph represents the mean optical density± SD. Statistical analysis was performed using one-way ANOVA followed by Bonferroni post-hoc test (*** *p* < 0.001). (**D**) Cell viability analysis after δ-TT treatment (20 μg/mL, 24 h) with or without Nec (50 μM) was performed in DU145 cells. Six independent biological samples for each condition were analyzed (*n* = 6); bar graph represents the mean relative cell viability ± SD. Statistical analysis was performed using one-way ANOVA followed by Bonferroni post-hoc test (* *p* < 0.05; *** *p* < 0.001). (**E**) Morphological analysis of DU145 cells after δ-TT treatment (20 μg/mL, 24 h) with or without Nec (50 μM) was analyzed by phase-contrast microscopy. (**F**) Cell viability analysis after δ-TT treatment (20 μg/mL, 24 h) with or without Nec (50 μM) was performed in PC3 cells. Six independent biological samples for each condition were analyzed (*n* = 6); bar graph represents the mean relative cell viability ± SD. Statistical analysis was performed using one-way ANOVA followed by Bonferroni post-hoc test (*** *p* < 0.001 vs. C; ** *p* < 0.01 vs. C). (**G**) Morphological analysis of PC3 cells after δ-TT treatment (20 μg/mL, 24 h) with or without Nec pretreatment (50 μM, 4 h) was analyzed by phase-contrast microscopy.

To analyze the impact of necroptosis on the antitumoral activity of δ-TT, we used Necrostatin-1 (Nec), a RIP1 kinase inhibitor widely used to inhibit necroptosis. At first, we performed flow cytometry analysis with Annexin V-FITC/PI double staining in DU145 and PC3 cells co-treated with δ-TT (20 μg/mL, 48 h) and Nec (50 μM) highlighting that necroptosis inhibitor counteracted the percentage of the cells in necrosis and late apoptosis (Figure 3A). Furthermore, we also analyzed the effect of Nec on RIP1 expression after δ-TT treatment. Figure 3B,C shows that Nec counteracted the δ-TT-induced RIP1 phosphorylation in both cell lines inhibiting the activation of RIP1-dependent necroptotic process. Based on the above results, we performed an MTT assay comparing treatment with δ-TT with the combination of δ-TT and Nec. The results obtained in both DU145 and PC3 cells demonstrated that the cytotoxic effect of δ-TT was counteracted by Nec (Figure 3D,F). Furthermore, the morphological analysis by phase-contrast microscopy confirmed the ability of Nec to revert the cellular alterations induced by δ-TT in DU145 (Figure 3E) and PC3 cells (Figure 3G).

All these results indicate that RIP1-dependent necroptosis triggered by δ-TT contributes to its cytotoxicity in both DU145 and PC3 cells.

### 2.4. δ-TT Potentiates Docetaxel Response in DU145 Cells and Counteracts Docetaxel-Resistance in DU-DXR Cells

DTX represents the first-line treatment for patients with CRCP; nevertheless, despite the prolonged survival, many patients developed chemoresistance [9].

Natural products are studied as promising sources of anticancer agents capable of inducing cell death alone or in association with other molecules [42].

The combined therapies with natural products and chemotherapeutic agents can often represent an opportunity to enhance the therapeutical response, avoiding the increase of side effects [38,43,44].

**Figure 4:**
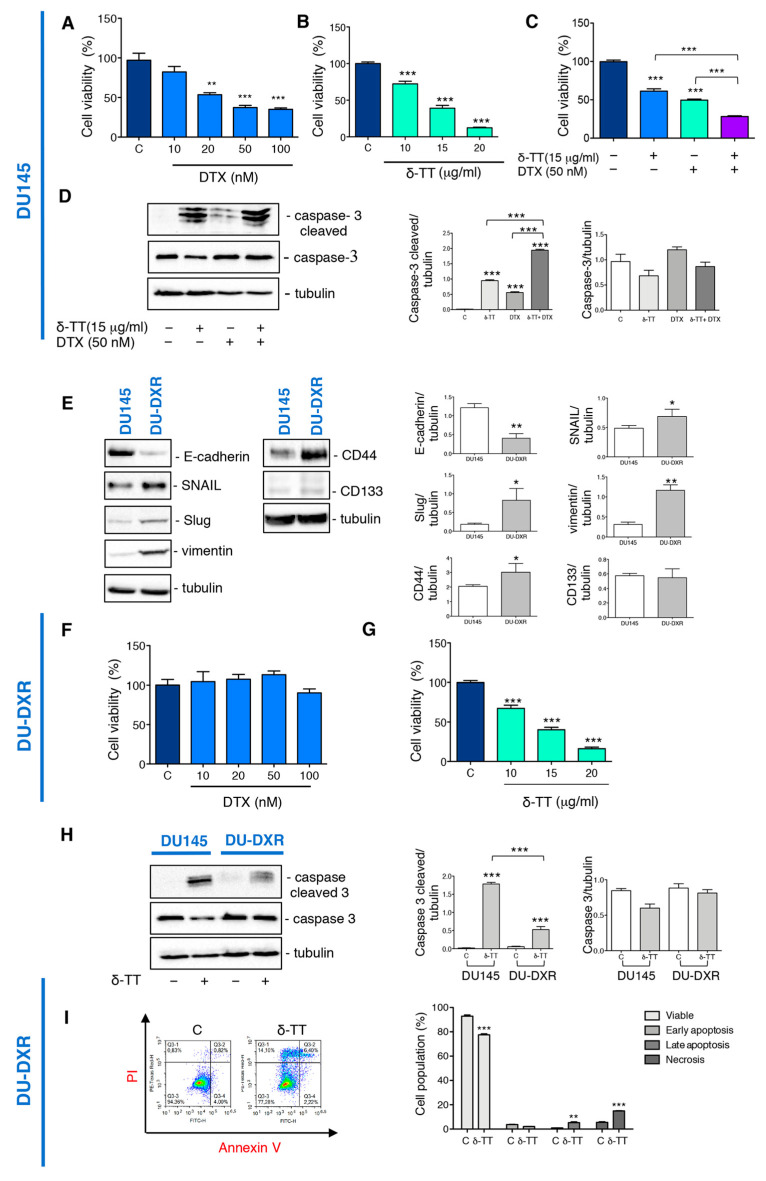
δ-TT potentiates docetaxel (DTX) response in DU145 cells and counteracts DTX-resistance in DU-DXR cells. (**A**) Cell viability analysis after DTX treatment (from 10 to 100 nM doses, 24 h) was determined by MTT assay in DU145 cells. Six independent biological samples for each condition were analyzed (*n* = 6); bar graph represents the mean relative cell viability ± SD. Statistical analysis was performed using one-way ANOVA followed by Dunnet post-hoc test (** = *p* < 0.01 vs. C; *** *p* < 0.001 vs. C). (**B**) Cell viability analysis after δ-TT treatment (from 10 μg/mL to 20 μg/mL, 24 h) was determined by MTT assay in DU145 cells. Six independent biological samples for each condition were analyzed (*n* = 6); bar graph represents the mean relative cell viability ± SD. Statistical analysis was performed using one-way ANOVA followed by Dunnet post-hoc test (*** *p* < 0.001 vs. C). (**C**) Cell viability analysis after δ-TT treatment (15 μg/mL, 24 h) in combination with or without DTX (50 nM, 24 h) was determined by MTT assay in DU145 cells. Six independent biological samples for each condition were analyzed (*n* = 6); bar graph represents the mean relative cell viability ± SD. Statistical analysis was performed using one-way ANOVA followed by Bonferroni post-hoc test (*** *p* < 0.001). (**D**) The expression of caspase-3 and caspase-3 cleaved were analyzed by Western blot after treatment with δ-TT (15 μg/mL, 48 h) in combination with or without DTX (50 nM, 48 h) in DU145 cells. Tubulin was used as loading control. Three independent experiments for each condition were analyzed; bar graph represents the mean optical density± SD. Statistical analysis was performed using one-way ANOVA followed by Bonferroni post-hoc test (*** *p* < 0.001). (**E**) The expression of E-cadherin, SNAIL, Slug, vimentin, CD44 and CD133 were analyzed by Western blot in both DU145 and DU-DXR cells. Tubulin was used as loading control. Three independent experiments for each condition were analyzed; bar graph represents the mean optical density ± SD. Statistical analysis was performed using Student’s test (* *p* < 0.05 vs. C; ** *p* < 0.01 vs. C). (**F**) Cell viability analysis after DTX treatment (from 10 to 100 nM doses, 24 h) was determined by MTT assay in DU-DXR cells. Six independent biological samples for each condition were analyzed (*n* = 6); bar graph represents the mean relative cell viability ± SD. Statistical analysis was performed using one-way ANOVA followed by Dunnet post-hoc test. (**G**) Cell viability analysis after δ-TT treatment (from 10 μg/mL to 20 μg/mL, 24 h) was determined by MTT assay in DU-DXR cells. Six independent biological samples for each condition were analyzed (*n* = 6); bar graph represents the mean relative cell viability ± SD. Statistical analysis was performed using one-way ANOVA followed by Dunnet post-hoc test (*** *p* < 0.001 vs. C). (**H**) The expression of caspase 3 and caspase-3 cleaved were analyzed by Western blot after treatment with δ-TT (20 μg/mL, 48 h) in both DU145 and DU-DXR cells. Tubulin was used as loading control. Three independent experiments for each condition were analyzed; bar graph represents the mean optical density± SD. Statistical analysis was performed using one-way ANOVA followed by Bonferroni post-hoc test (*** *p* < 0.001). (**I**) Flow cytometric analysis by Annexin-FITC/PI double staining was performed after δ-TT treatment (20 μg/mL, 48 h) in DU-DXR cells. Three independent biological samples for each condition were analyzed (*n* = 3); bar graph represents the mean ± SD. Statistical analysis was performed using Student’s test (** *p* < 0.01 vs. C; *** *p* < 0.001 vs. C).

We then analyzed whether δ-TT treatment could sensitize the DTX response by cell viability assay. Preliminary experiments demonstrated that DTX treatment for 24 h at doses from 10 nM to 100 nM induce a significant reduction in the viability of DU145 cells (Figure 4A), while δ-TT induced a cytotoxic effect from 10 to 20 μg/mL (24 h) (Figure 4B). The combined treatment with the two substances (δ-TT 15 μg/mL and DTX 50 nM for 24 h) show an enhancement of the cytotoxic response compared to the single substances (Figure 4C). To better understand the mechanism by which this enhancement happened, we analyzed the caspase 3 activation. Figure 4D shows that δ-TT (15 μg/mL, 48 h) activated the cleavage of caspase 3, as well as DTX (50 nM, 48 h). The combination of the two substances significantly increased the levels of cleaved caspase 3 without changing caspase 3 expression (Figure 4D).

It is, therefore, possible that δ-TT, through its ability to activate apoptosis, could enhance the response to DTX suggesting its possible use in combination with chemotherapy and simultaneously to reduce the side effects associated to it.

To also investigate a possible use of δ-TT when resistance to DTX develops, we created a DTX-resistant cell line (DU-DXR) derived from the DU145 cell line. First, we characterized this cell line by analyzing the molecular markers involved in the process of resistance to DTX.

EMT is a phenomenon associated with drug resistance [45], and often cells that have undergone EMT become resistant to apoptosis and senescence. EMT induction is characterized by suppression of the expression of epithelial markers, such as E-cadherin, and the induction of mesenchymal markers, such as N-cadherin and vimentin [46].

We have, therefore, analyzed the expression of EMT markers in DU-DXR. WB analysis revealed that DU-DXR cells were characterized by downregulated expression of E-cadherin and upregulated expression of vimentin compared to parental DU145 (Figure 4E). In addition, since transcription factors, such as Snail and Slug, are closely correlated with EMT and acquired drug resistance [19,46,47], we have evaluated their expression in DU-DXR cells compared to DU145. The WB analysis shown in Figure 4E revealed a major expression of both transcription factors in DU-DXR cells compared to DU145 parental cells.

Moreover, we also detected the expression level of CD44 and CD133 because drug resistance was linked to the presence of CSC [20,21]. WB analysis revealed that DU-DXR presented higher expression levels of CD44 compared to DU145 parental cells, whereas CD133 protein was not expressed in both cell lines (Figure 4E).

Then, to confirm the acquisition of DTX-resistance, we analyzed the cell viability of DU-DXR cells after DTX treatment observing that DTX does not reduce viability at any doses considered (Figure 4F).

The experiments described above, therefore, allow us to consider DU-DXR cell line as a good cellular model to evaluate the acquisition of DTX- resistance.

Therefore, we investigated whether the treatment with δ-TT induced cell death in DU-DXR cells. MTT assays demonstrated that δ-TT (10, 15 and 20 μg/mL, 24 h) significantly reduced DU-DXR viability in a dose-dependent manner similar to that in DU145 cells (Figure 4G).

Analyzing the effect of δ-TT treatment (20 μg/mL, 48 h) on cleaved caspase 3 expression, we observed that δ-TT treatment was not able to induce a significant activation of caspase 3 in DU-DXR compared to DU145 parental cells (Figure 4H). This finding suggested that in cells that have developed resistance to apoptosis, δ-TT maintained cellular cytotoxicity activating a non-apoptotic alternative cell death.

Hence, we performed flow cytometry analysis in DU-DXR after δ-TT treatment (20 μg/mL, 48 h). The results shown in Figure 4I demonstrate an increase in necrosis (Annexin V^−^/PI^+^) (14.10%) and late apoptosis (Annexin V^+^/PI^+^) (6.4%) in the treated sample.

Overall, the results obtained demonstrate that the δ-TT induces cytotoxicity in DTX-resistant cells, involving a necrotic event and only slight apoptosis.

### 2.5. δ-TT Induced Necroptosis in DU-DXR Prostate Cancer Cells

**Figure 5:**
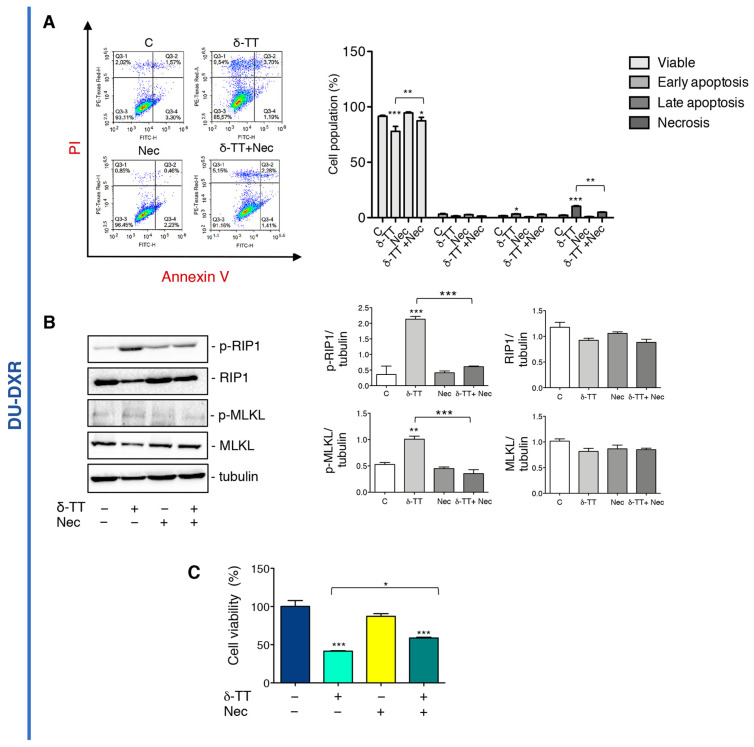
δ-TT induced necroptosis in DU-DXR prostate cancer cells. (**A**) Flow cytometric analysis by Annexin-FITC/PI double staining was performed after δ-TT treatment (20 μg/mL, 48 h) in combination with Nec (50 μM) in DU-DXR cells. Three independent biological samples for each condition were analyzed (*n* = 3); bar graph represents the mean ± SD. Statistical analysis was performed using one-way ANOVA followed by Bonferroni post-hoc test (* *p* < 0.05; ** *p* < 0.01; *** *p* < 0.001). (**B**) The effect of δ-TT (20 μg/mL, 24 h) in presence of Nec (50 μM) was evaluated on p-RIP1, RIP1, p-MLKL and MLKL expression in DU-DXR cells. Tubulin was used as loading control. Three independent experiments for each condition were analyzed; bar graph represents the mean optical density± SD. Statistical analysis was performed using one-way ANOVA followed by Bonferroni post-hoc test (** *p* < 0.01; *** *p* < 0.001). (**C**) Cell viability analysis after δ-TT treatment (20 μg/mL, 24 h) with or without Nec (50 μM) was performed in DU-DXR cells. Six independent biological samples for each condition were analyzed (*n* = 6); bar graph represents the mean relative cell viability ± SD. Statistical analysis was performed using one-way ANOVA followed by Bonferroni post-hoc test (* *p* < 0.05; *** *p* < 0.001).

To further investigate if δ-TT-induced necrosis in DU-DXR cells could be necroptosis, cells were incubated with Nec in association with δ-TT. The flow cytometry analysis confirms that the number of cells in δ-TT-induced necrosis were reduced in the presence of Nec (Figure 5A). Moreover, WB experiments showed that δ-TT increases both RIP1 and MLKL phosphorylation, whereas Nec markedly counteracts these events (Figure 5B). This result confirms that even in DTX-resistant cells, δ-TT induces RIP1-dependent necroptosis. In addition, MTT analysis showed that δ-TT cytotoxicity was significantly counteracted by Nec (Figure 5C) demonstrating that in DU-DXR cells δ-TT treatment induces cell death through the activation of necroptosis.

## 3. Discussion

DTX is the first-line therapy for patients with metastatic CRPC, but acquisition of drug resistance due to evasion and defection of apoptosis often leads to the failure of this therapeutical approach [21].

Natural products are emerging as a promising source for effective anticancer agents and are often less toxic than chemotherapeutic agents. Additionally, they can activate molecular mechanisms able to contrast resistance to therapies [44].

In this work, we investigated whether the cytotoxic activity of δ-TT in CRPC cells was due to the activation of necroptosis, an alternative form of RCD, in addition to the ability to induce apoptosis involving ER stress and autophagy [34].

The results obtained confirmed that δ-TT actives necroptotic machinery promoting RIP1 and MLKL phosphorylation and MLKL translocation into the membranes, as well as decreasing FLIP_L_ expression by preventing caspase-8 activation in both DU145 and PC3 cells. In addition, we have shown that δ-TT-induced necroptosis contributes to cell death. The obtained results demonstrate for the first time that the δ-TT can activate an alternative form of cell death, which can be effective when tumor cells undergo molecular modifications that alter the ability to respond to apoptotic stimuli.

The ability of the δ-TT to induce necroptosis adds to many previous studies, which reported that different natural products, such as green tea extracts [48], shikonin [49,50], curcumin [51], celastrol [52], bufalin [53], berberine [54], resveratrol [55], goniothalamin [56], latifolin [57] and others, are able to induce necroptosis [36]. The ability of δ-TT to induce necroptosis in CRPC cells prompted us to investigate whether δ-TT was able to increase cell death in association with DTX. In fact, combination therapy with natural products and chemotherapy is thought to enhance anticancer actions through simultaneous targeting of multiple pathways [44].

Then, cell viability was analyzed in DU145 cells treated with δ-TT in combination with DTX demonstrating that δ-TT potentiated DTX action. In line with our data, other authors demonstrated that: (1) γ-TT enhances chemosensitivity to DTX on oral cancer cells [58]; (2) a nanoemulsion of tocotrienols and caffeine synergistically potentiated cisplatin action on hepatocarcinoma cells, preserving normal cells to side effects of chemotherapy [59]; and (3) δ-TT sensitizes ovarian cancer cells to cisplatin [60].

One of the unsolved problems of chemotherapy is the development of resistance mainly due to the selection of cells with genetic and phenotypic modifications, which make them resistant to death by apoptosis.

We then analyzed the effect of δ-TT on CRPC cells resistant to DTX highlighting that, also in these cells, the drug exerted a cytotoxic effect without activating a canonical apoptotic death but activating the necroptotic process.

Necroptosis induction could, indeed, represent an alternative therapeutical approach for apoptosis resistant CRPC.

Also, previously Asay and collaborators demonstrated that γ-TT and α-tocopherol enhanced DTX activity in drug-resistant PC3 cells suggesting the use of these molecules to treat prostate cancer in combination with DTX and to ameliorate therapeutic resistance [61].

In conclusion, our study demonstrated that δ-TT exerts its antitumoral activity in CRPC activating the classic programmed apoptotic death but also by triggering an alternative programmed death, such as necroptosis. The activation of this process allows us to overcome DTX chemoresistance caused by the evasion of apoptotic pathways.

Our findings provided a basis for considering δ-TT as potential adjuvant therapy for the treatment of metastatic DTX-resistant CRPC.

## 4. Materials and Methods

### 4.1. Reagents and Antibodies

The δ-TT was purified from a commercial extract of Annatto (*Bixa orellana* L.) seeds (American River Nutrition Inc., Hadley, MA, USA) as previously described [62]. Docetaxel (DTX), necrostatin-1 (Nec), dimethyl sulfoxide (DMSO) and 3-(4,5)-dimethylthiazol-2-yl-2,5-diphenyltetrazolium bromide (MTT) were purchased from Sigma-Aldrich (St. Louis, MO, USA). The pan-caspase inhibitor carbobenzoxy-valyl-alanyl-aspartyl-[O-methyl]- fluoromethylketone (Z-VAD) was from R&D System Inc. (Minneapolis, MN, USA). Annexin V-FITC/PI apoptosis detection kit was from eBioscience (Vienna, Austria).

The primary antibody against RIP1 (D94C12) (#3493), p-RIP1 (Ser166) (D1L3S) (#65746), MLKL (D2I6N) (#14993), p-MLKL (Ser358) (D6H3V) (#91689), caspase-8 (D35G2) (#4790), cleaved-caspase-8, caspase-3 (#9665), cleaved-caspase-3 (Asp-175) (5A1E) (#9664), E-cadherin (#3195), Snail (#3879), Slug (#9585), vimentin (#5741), CD44 (#3570), rabbit and mouse horseradish-peroxidase-conjugated secondary antibody were from Cell Signaling Technology Inc. (Boston, MA, USA); CD133 (# MAB4399) was from Millipore (Burlington, MA, USA). Alpha-tubulin (T6199) was from Sigma-Aldrich; MLKL (3B2) (sc-293201) and FLIP_S/L_ (G-11) (sc-5276) were from Santa Cruz Biotechnology Inc. (Heidelberg, Germany).

### 4.2. Cell Culture

Two human prostate cancer cell lines (DU145 and PC3) were purchased from American Type Culture Collection ATCC, Manassas, VA, USA). The cells were authenticated by Short Tandem Repeat (STR) analysis as described in ANSI Standard (ASN-0002) (ATCC Standards Development Organization). Both cell lines were maintained in RPMI-1640 medium (EuroClone, Milano, Italy) supplemented with 5% (DU145) and 7.5% (PC3) Fetal Bovine Serum (FBS) (Gibco, ThermoFisher Scientific, Waltham, MA, USA), glutamine (1 mmol/L) and antibiotics (100 IU/mL penicillin G) and cultured at 37 °C in humidified atmosphere of 5% CO_2_.

### 4.3. Induction of DTX-Resistant DU145 Cells

To generate DTX-resistant cells, DU145 cells were chronically exposed to increasing concentrations of DTX (5, 10, 20, 50, 100, 200 nM) in 75 cm^2^ flasks for 48 h. After treatment, the surviving cells were seeded into a new flask and grown for 2–3 weeks. The cells that survive after 2 treatment cycles with DTX 200 nM were designated as DU-DXR. DU-DXR, developed by stepwise increased concentration of DTX over a period of 6 months, were then continuously maintained in complete RPMI-1640 medium supplemented with 5 nM DTX.

Resistance was judged on decreased cell death and increased proliferation analyzed by cell viability assay.

### 4.4. Cell Viability Assay

DU145, PC3 and DU-DXR were seeded in 24-well culture plates at a concentration of 3 × 10^4^ cells/well. DU145 and PC3 were treated with δ-TT (20 μg/mL, corresponding to 50 μM) for 24 h with or without pre-incubation with Z-VAD (50 μM) or Nec (50 μM) for 4 h. DU145 and DU-DXR were treated with different concentration of DTX (10, 20, 50, 100 and 200 nM) or δ-TT (10, 15, 20 μg/mL, corresponding to 25.2 μM, 37,8 μM and 50 μM). DU-DXR were also treated with δ-TT (20 μg/mL,) for 24 h with or without a pre-incubation with Nec (50 μM) for 4 h. After different treatments, MTT solution (0.5 mg/mL) in RPMI-1640 without phenol red and FBS was added to each well and incubated at 37 °C for 15 min (DU145 and DU-DXR) or 45 min (PC3). Subsequently, culture media were removed and replaced with isopropanol to dissolve the crystals. The OD values were measured at wavelength of 550 nm through an EnSpire Multimode Plate reader (PerkinElmer, Milano, Italy). Each experiment was repeated three times.

### 4.5. Cell Morphological Analysis

DU145 and PC3 were seeded in 24-well culture plates at a concentration of 3 × 10^4^ cells/well. Cells were treated with δ-TT (20 μg/mL) for 24 h with or without a pre-incubation with Z-VAD (50 μM) or Nec (50 μM) for 4 h. Morphological analysis was performed by optical microscopy from different fields under a Zeiss Axiovert 200 microscope with a 20 × 0.4 objective lens linked to a CoolSnap Es CCD camera (Roper Scientific-Crisel Instruments, Rome, Italy).

### 4.6. Cell Death Analysis by Flow Cytometry

Cells were seeded in 6 cm Petri plate at a density of 1.5 × 10^5^ cells/plate for 24 h and then treated for 48 h with δ-TT (20 μg/mL) alone or following a pre-treatment with Nec (50 μM) for 4 h. After treatment, the cells were harvested, washed with phosphate buffer solution (PBS), resuspended in binding buffer (BB) 1X and incubated with Annexin V-FITC/PI according to the manufacturer’s instructions. The stained cells were analyzed by flow cytometry Novocyte 3000 (Acea Bioscience, Inc., San Diego, CA, USA) and results analyzed by software Novo Express (Version 1.4.1). Each experiment was repeated three times.

### 4.7. Western Blot Assay

DU145, DU-DTX and PC3 cells were seeded at 1.5 or 2.5 × 10^5^ cells/dish in 6 cm Petri dishes. After each treatment, adherent and floating cells were harvested and lysed in RIPA buffer (0.05 mol/L Tris. HCl pH 7.7, 0.15 mol/L NaCl, 0.8% SDS, 10 mmol/L EDTA, 100 μmol/L NaVO4, 50 mmol/L NaF, 0.3 mmol/L PMSF, 5 mmol/L iodoacetic acid) containing leupeptin (50 μg/mL), aprotinin (5μL/mL) and pepstatin (50 μg/mL); protein preparations (25–35 μg) were resolved on SDS-PAGE and transferred to nitrocellulose membranes. After 1 h in blocking buffer, membranes were incubated overnight at 4 °C with the specific primary antibody. Tubulin was utilized as a loading control. Detection was done using horseradish peroxidase-conjugated secondary antibodies and enhanced chemiluminescence kit Westar Etac Ultra 2.0 (XLS075,0100) (Cyanagen, Bologna Italy).

### 4.8. Membrane Proteins Extraction

Cells were seeded in 6 cm Petri plate at a density of 1.5 × 10^5^ cells/plate for 24 h and then treated for 24 h with δ-TT (20 μg/mL).

The membrane proteins extraction was performed using Mem-PER Plus Membrane Protein Extraction Kit (Thermo Fisher Scientific, Waltham, MA, USA) according to manufacturer’s protocol.

This kit ensures the extraction of membrane (plasma and internal) proteins, and the contamination of cytosolic proteins into the membrane fraction is usually less than 10%.

### 4.9. Immunofluorescence Assay

DU145 and PC3 cells were plated onto polylysine-coated coverslips in 24-well culture plates at a concentration of 3 × 10^4^ cells/well. After treatment with δ-TT (20 μg/mL) for 24 h, the cells were washed with PBS three times and subsequently fixed with 4% paraformaldehyde/1% sucrose solution for 10 min at room temperature (RT). Following three washes in PBS, the cells were then permeabilized with PBS containing 0.5% Triton X-100 for 20 min at RT. The cells were washed thrice in PBS for 5 min each and then blocked with horse serum solution for 20 min at RT. The cells were then incubated with anti-MLKL antibody overnight at 4 °C. The next day, cells were incubated with FITC-labeled anti-mouse secondary antibody for 1 h in the dark at RT. Finally, the cells were incubated with the fluorescent stain 4′,6-diamidino-2-phenylindole, DAPI for another 15 min. After staining, coverslips were rinsed and mounted in Mowiol onto slides and data acquisition and image processing were performed by a Zeiss Axiovert 200 microscope with a 63 × 1.4 objective lens linked to a Coolsnap Es CCD camera (Roper Scientific-Crisel Instruments, Roma, Italy) using MetaMorph^®^ (MetaMorph Inc., Nashville, TN, USA).

### 4.10. Statistical Analysis

All experiments were performed three times and the results were analyzed by unpaired Student’s *t*-test or by one-way analysis of variance (ANOVA) followed by Dunnet’s or Bonferroni’s post-test using the Prism software (Prism 8 for Mac OS version 8.2.1, GraphPad Software San Diego, CA, USA).

## Data Availability

All data are presented in the manuscript and Supplementary Materials.

## Supplementary Materials

The supporting information can be downloaded at: https://www.mdpi.com/article/10.3390/ijms24054923/s1.
