# Supplementary material for: Necroptosis Induced by Delta-Tocotrienol Overcomes Docetaxel Chemoresistance in Prostate Cancer Cells
Source: Int J Mol Sci. 2023 Mar 3;24(5):4923. doi: 10.3390/ijms24054923 (PMC10003232; doi:10.3390/ijms24054923)
Supplement: Supplementary file 1 [file ijms-24-04923-s001.zip › ijms-2210273-supplementary.pdf]

Figure 1E

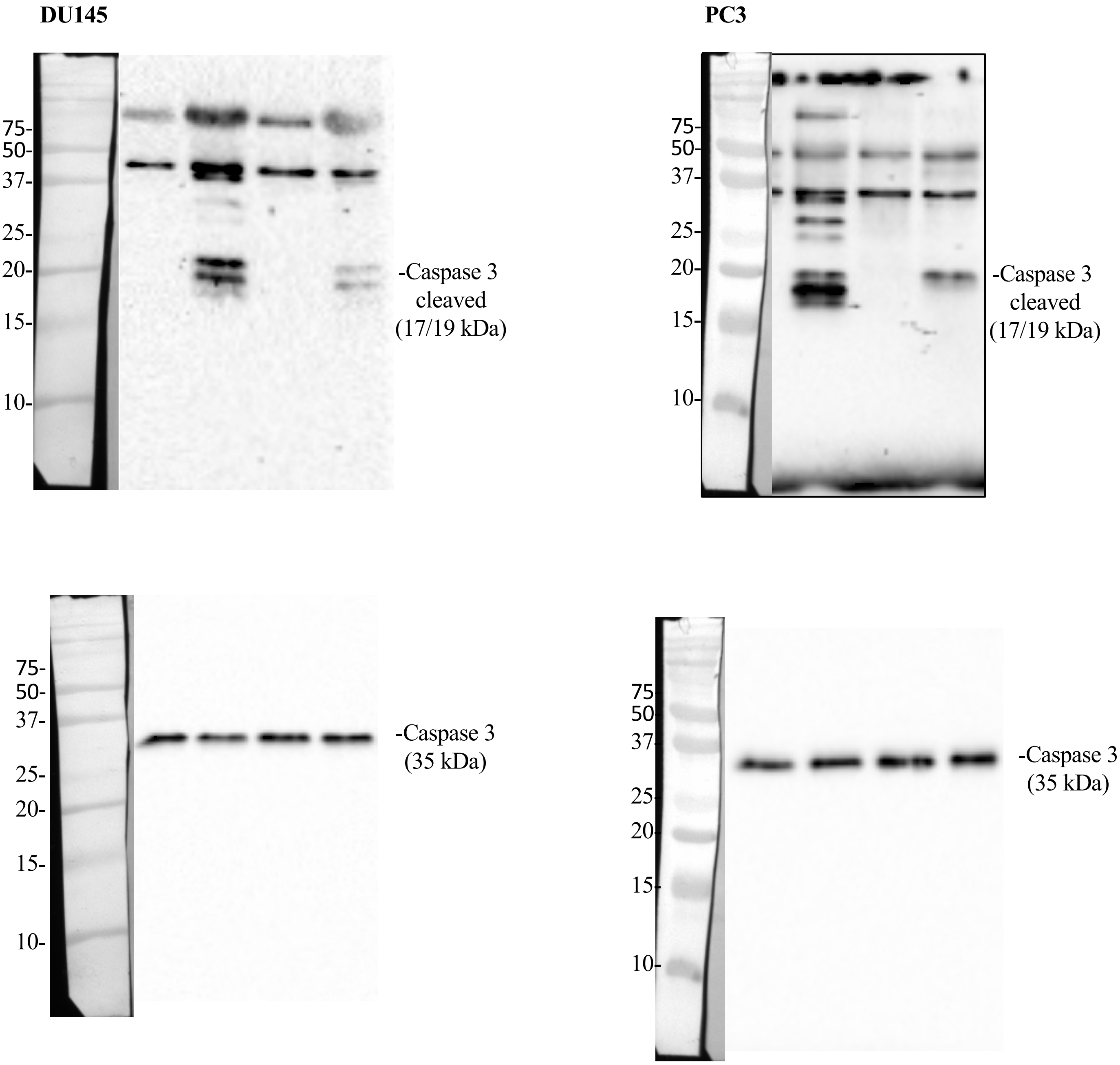
DU145
PC3
75-
50-
37-
25-
20-
15-
10-
-Caspase 3 cleaved (17/19 kDa)
-Caspase 3 (35 kDa)

Figure 2A

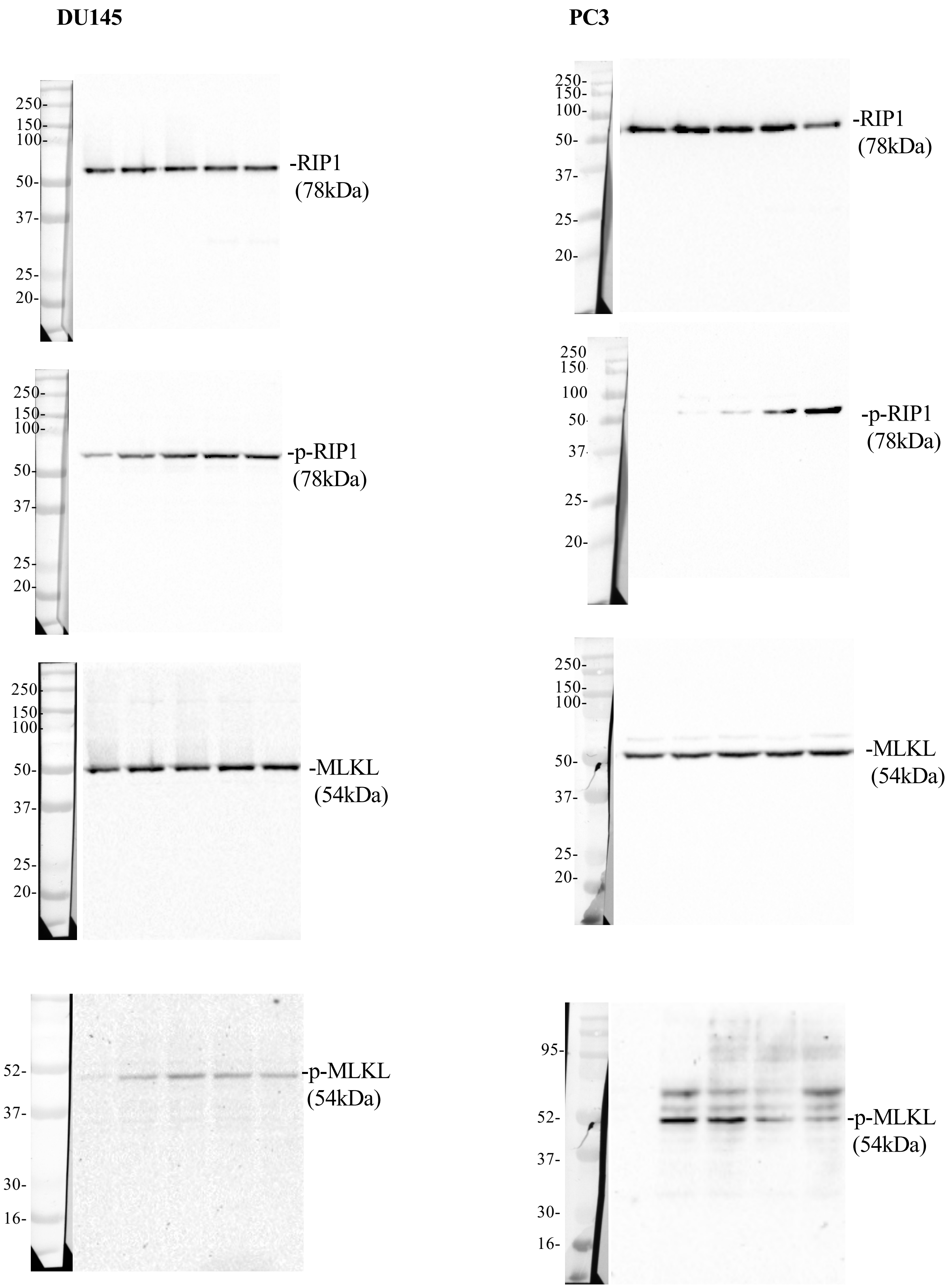
DU145
PC3
250-
150-
100-
50-
37-
25-
20-
-RIP1
(78kDa)
-p-RIP1
(78kDa)
-MLKL
(54kDa)
52-
37-
30-
16-
95-
-p-MLKL
(54kDa)

Figure 2B

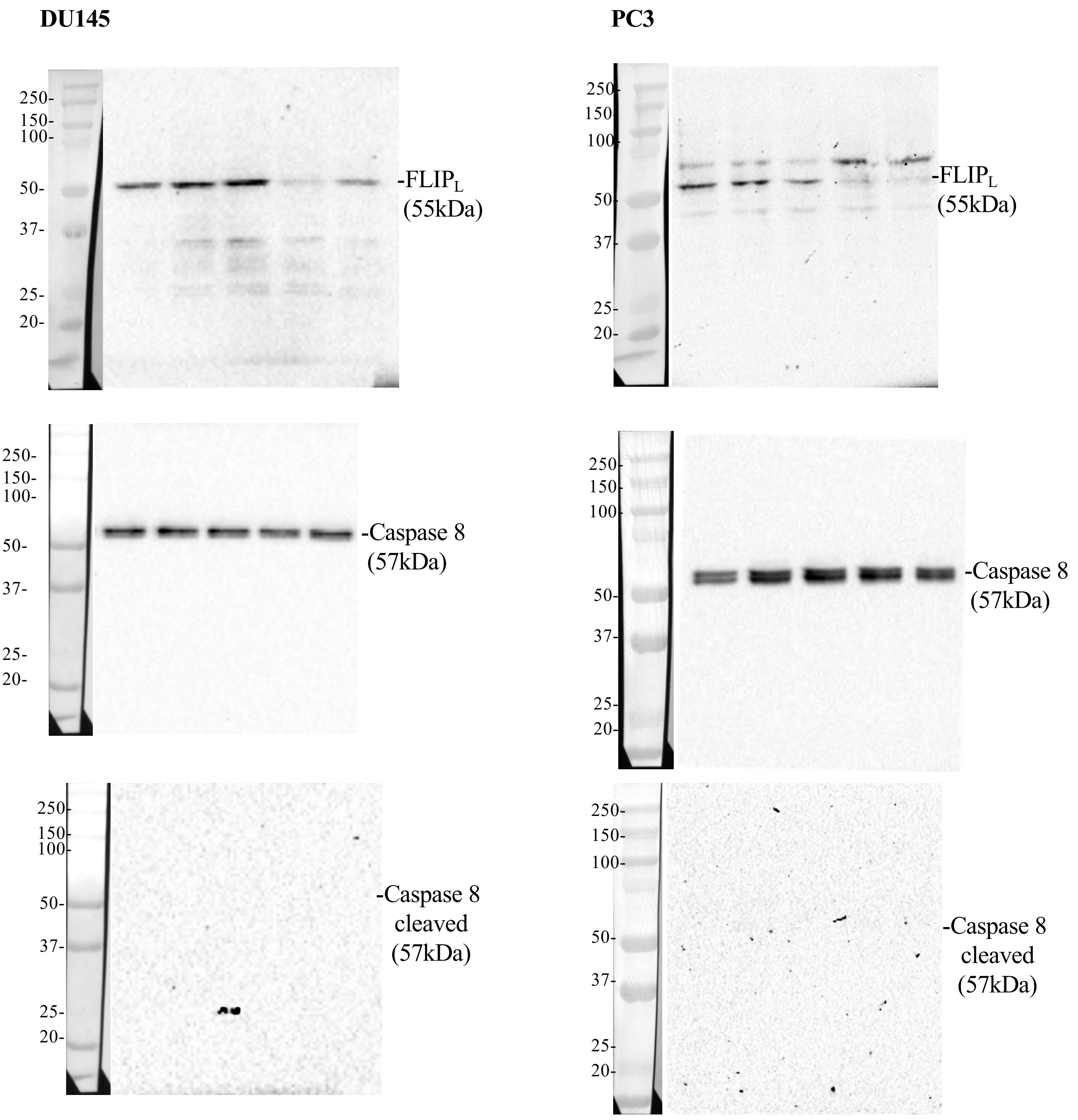

Figure 2C

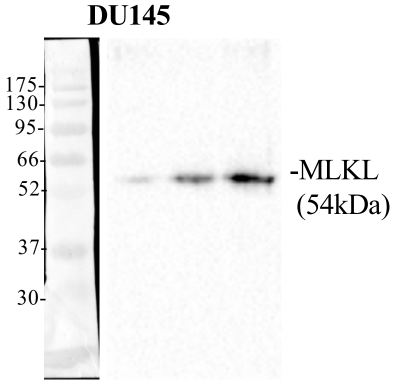

Figure 2E

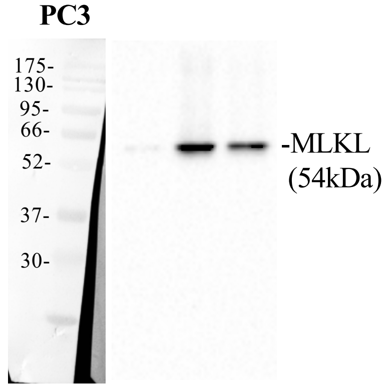

Figure 3B

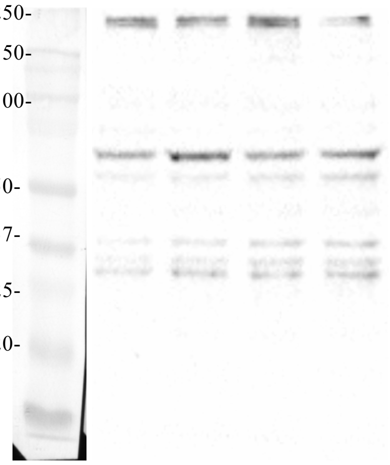

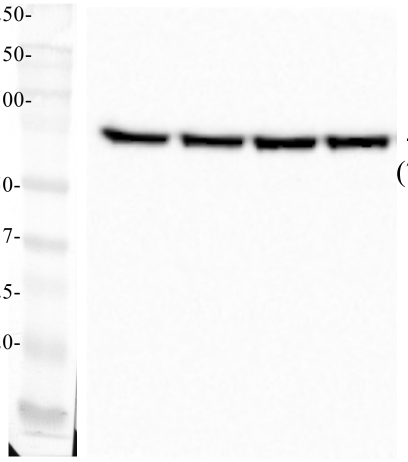

Figure 3C

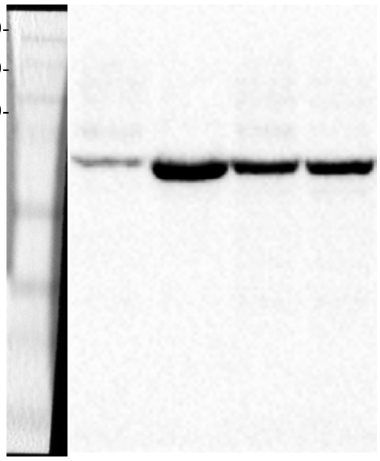

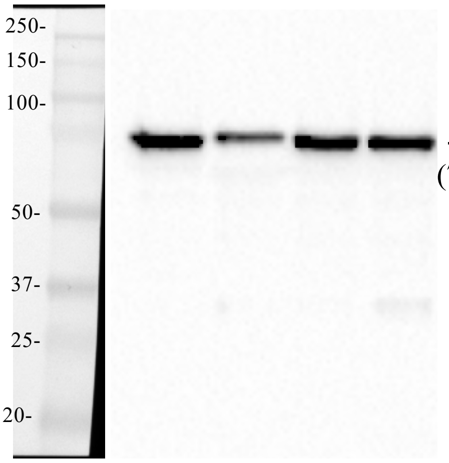

Figure 4D

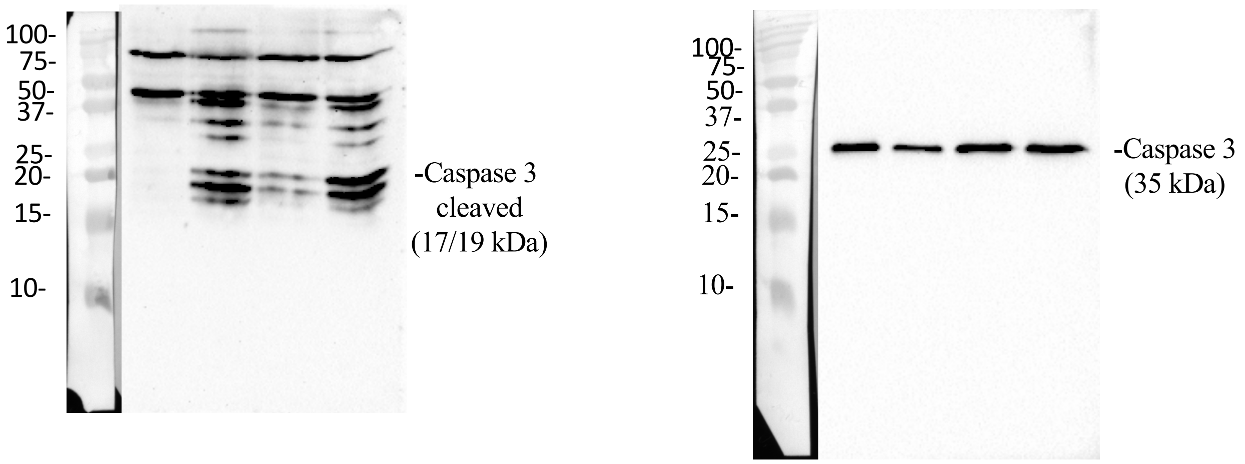

Figure 4E

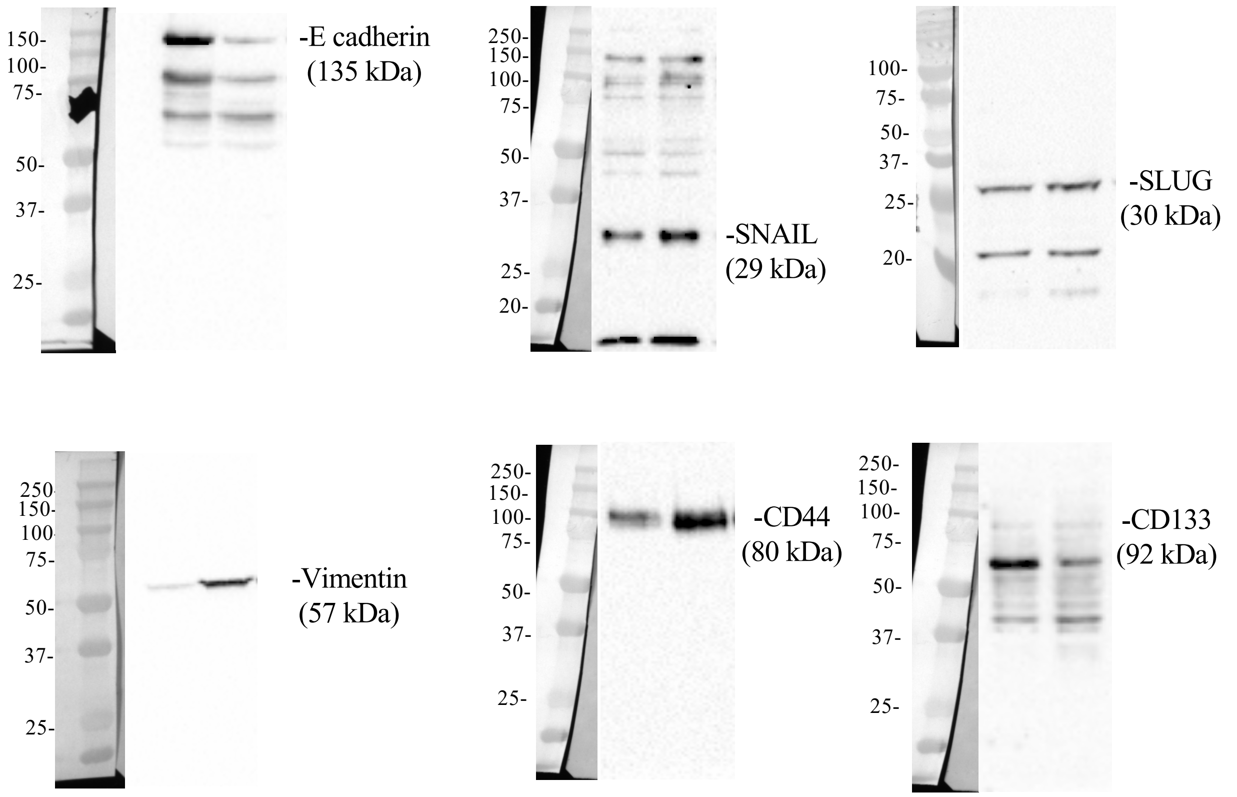

Figure 4H

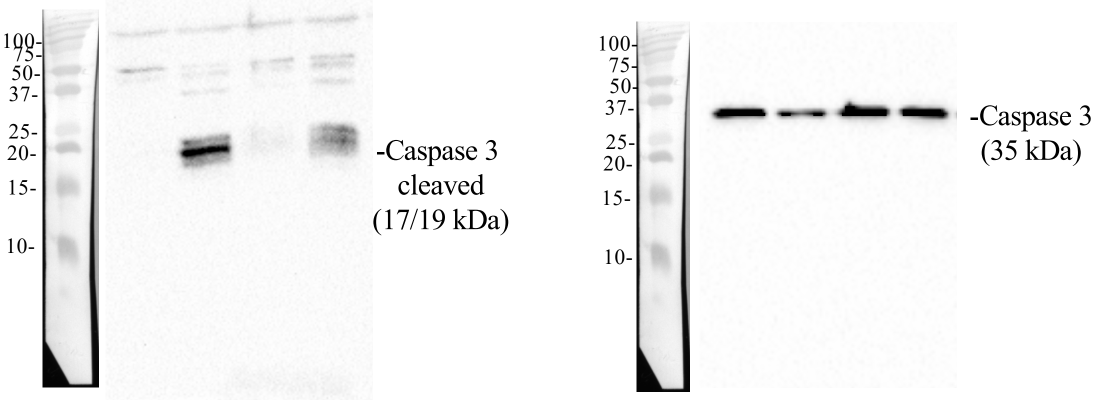

Figure 5B

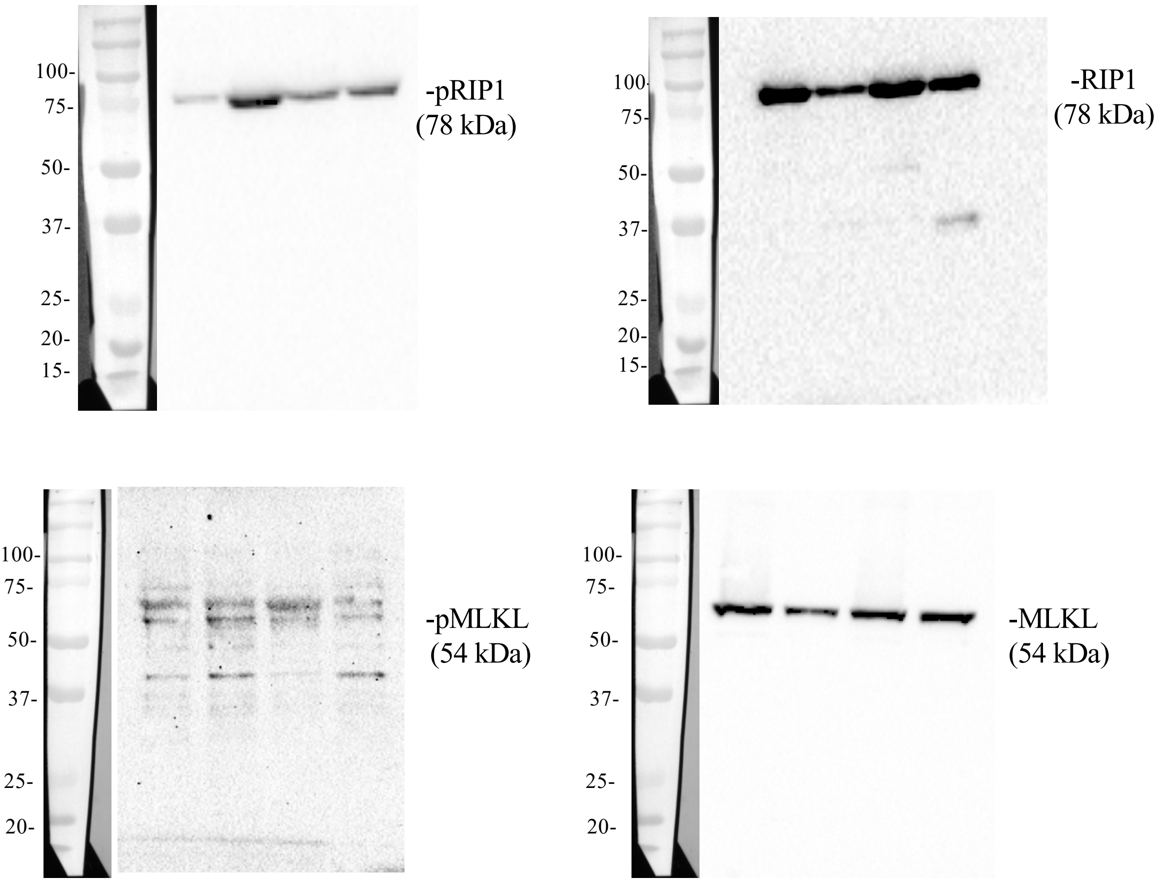

100-
75-
50-
37-
25-
20-
15-
-pRIP1
(78 kDa)
100-
75-
50-
37-
25-
20-
15-
-RIP1
(78 kDa)
100-
75-
50-
37-
25-
20-
-pMLKL
(54 kDa)
100-
75-
50-
37-
25-
20-
-MLKL
(54 kDa)
